# Defining a metagenomic threshold for detecting low abundances of *Providencia alcalifaciens* in canine faecal samples

**DOI:** 10.3389/fcimb.2024.1305742

**Published:** 2024-02-28

**Authors:** Anja Maria Aardal, Eiril Moen Soltvedt, Simen Foyn Nørstebø, Thomas H. A. Haverkamp, Sabrina Rodriguez-Campos, Ellen Skancke, Ann-Katrin Llarena

**Affiliations:** ^1^ Bacteriology and Mycology Unit, Department of Paraclinical Sciences, Faculty of Veterinary Medicine, Norwegian University of Life Sciences, Ås, Norway; ^2^ Norwegian Veterinary Institute, Ås, Norway; ^3^ Small Animal Section, Department of Companion Animal Clinical Sciences, Faculty of Veterinary Medicine, Norwegian University of Life Sciences, Ås, Norway; ^4^ Food Safety Unit, Department of Paraclinical Sciences, Faculty of Veterinary Medicine, Norwegian University of Life Sciences, Ås, Norway

**Keywords:** shotgun sequencing, faecal microbiota, AHDS, canine, detection limit, clinical metagenomics

## Abstract

**Introduction:**

Acute haemorrhagic diarrhoea syndrome (AHDS) in dogs is a condition of unknown aetiology. *Providencia alcalifaciens* is suspected to play a role in the disease as it was commonly found in dogs suffering from AHDS during a Norwegian outbreak in 2019. The role of this bacterium as a constituent of the canine gut microbiota is unknown, hence this study set out to investigate its occurrence in healthy dogs using metagenomics.

**Materials and methods:**

To decrease the likelihood of false detection, we established a metagenomic threshold for *P. alcalifaciens* by spiking culture-negative stool samples with a range of bacterial dilutions and analysing these by qPCR and shotgun metagenomics. The detection limit for *P. alcalifaciens* was determined and used to establish a metagenomic threshold. The threshold was validated on naturally contaminated faecal samples with known cultivation status for *P. alcalifaciens*. Finally, the metagenomic threshold was used to determine the occurrence of *P. alcalifaciens* in shotgun metagenomic datasets from canine faecal samples (n=362) collected in the HUNT One Health project.

**Results:**

The metagenomic assay and qPCR had a detection limit of 1.1x10^3^ CFU *P. alcalifaciens* per faecal sample, which corresponded to a Cq value of 31.4 and 569 unique *k-*mer counts by shotgun metagenomics. Applying this metagenomic threshold to 362 faecal metagenomic datasets from healthy dogs, *P. alcalifaciens* was found in only 1.1% (95% CI [0.0, 6.8]) of the samples, and then in low relative abundances (median: 0.04%; range: 0.00 to 0.81%). The sensitivity of the qPCR and shotgun metagenomics assay was low, as only 40% of culture-positive samples were also positive by qPCR and metagenomics.

**Discussion:**

Using our detection limit, the occurrence of *P. alcalifaciens* in faecal samples from healthy dogs was low. Given the low sensitivity of the metagenomic assay, these results do not rule out a significantly higher occurrence of this bacterium at a lower abundance.

## Introduction

An outbreak of canine acute haemorrhagic diarrhoea syndrome (AHDS) occurred in Southeast Norway during the autumn of 2019, resulting in several cases of severe illness and deaths (Haaland et al., 2020; Jørgensen et al., 2021). AHDS is characterized by rapid onset of haemorrhagic diarrhoea, sometimes accompanied by vomiting (Heilmann et al., 2017), followed by rapid deterioration, shock and death if left untreated (Trotman, 2015). The aetiology of the condition is unknown; however, it has been suggested that dietary components and bacterial toxins play a role (Hall and Day, 2017). The bacterium *Clostridium perfringens* and its toxins, particularly NetF, have previously been associated with the condition (Leipig-Rudolph et al., 2018; Sindern et al., 2019; Busch and Unterer, 2022), albeit the presence of toxin genes could not explain all findings. *Providencia alcalifaciens* is a Gram-negative, rod-shaped facultative anaerobe in the family *Morganellaceae* (Adeolu et al., 2016). It has previously been identified in haemorrhagic diarrhoea in a litter of puppies (Möhr et al., 2002) and haemorrhagic pneumonia in piglets (Wang et al., 2014), and it was suspected as a causative agent of canine enteritis in Norway in 2005 (Fauske and Hirsch, 2006). Indeed, during the outbreak in 2019, *P. alcalifaciens* was found in the faeces of more than half the dogs (62%) admitted to the Small Animal Hospital at the Norwegian University of Life Sciences with AHDS (Haaland et al., 2020). Further, non-culture-based methods revealed an increase in the relative abundance of *P. alcalifaciens* in faeces from dogs suffering from AHDS (Herstad et al., 2021). The bacterium was rarely found in healthy controls (Jørgensen et al., 2021). Therefore, *P. alcalifaciens* is suspected to play a role in the aetiology of AHDS.

Bacterial diagnostics is traditionally carried out using cultivation, but cultivation is time-consuming and unsuitable for unculturable bacteria. Culture-independent methods, such as qPCR, are being increasingly utilized in diagnostic bacteriology, especially in public health laboratories (Huang et al., 2016), but also in veterinary diagnostics (Toohey-Kurth et al., 2020). However, qPCR has the drawback of only investigating a limited number of known targets per assay (Blauwkamp et al., 2019), and leaves no isolate for further in-depth studies by e.g. sequencing. Investigating the entire bacteriological content and pathological potential of a sample simultaneously is therefore an alluring option. Here, amplicon sequencing and shotgun metagenomic sequencing have the potential to identify thousands of microbial species in the same sample. Indeed, metagenomic approaches to reach an aetiological diagnosis of infection have been developed and used to detect pathogens in cerebrospinal fluid (Miller et al., 2019; Morsli et al., 2022), faeces (Nakamura et al., 2008) and blood (Blauwkamp et al., 2019), as well as other biological specimens as reviewed by Forbes et al. (2018). Stool is, however, a complex matrix, as it contains a myriad of microorganisms, of which most are harmless or beneficial commensal microorganisms (Chiu and Miller, 2019). Identifying and interpreting the presence of low-abundance pathogens in this complex ecosystem is challenging, as the presence of more abundant species may overshadow the rare species. The sensitivity of shotgun metagenomics in detecting low-abundance bacteria in stool samples will therefore depend on sequencing depth, the complexity of the sample, and the abundance threshold for detection. Setting an abundance threshold is especially cumbersome; if the threshold is set too high, low-abundance bacteria may be missed, while setting a lower threshold may increase the number of false-positive samples. The choice of the threshold will therefore depend on the specific research question and the desired level of sensitivity. These factors will vary between pathogenic targets as well as matrix under investigation. Further, misclassifications of sequences are commonly encountered, especially when using deep sequencing, as shown in several studies where metagenomic classifiers readily identify hundreds of species in known mock communities despite only a few being present (Haverkamp et al., 2021). Additional sources of misclassifications include contamination, presence of kitome, index hopping and sample bleeding (Mitra et al., 2015; van der Valk et al., 2020).

Here, we examined the concordance, sensitivity, and specificity of bacterial culture, qPCR, and shotgun metagenomics to detect *P. alcalifaciens* in spiked and naturally contaminated stool samples from dogs, with the aim of setting a suitable abundance threshold for shotgun metagenomics. Further, we used the shotgun metagenomics assay to investigate the occurrence of *P. alcalifaciens* in a large dataset of healthy dogs, informing on the occurrence of this bacterium in the core microbiota in canine faeces. This knowledge is needed to evaluate the role of *P. alcalifaciens* in canine AHDS.

## Materials and methods

### Study design

**Figure 1:**
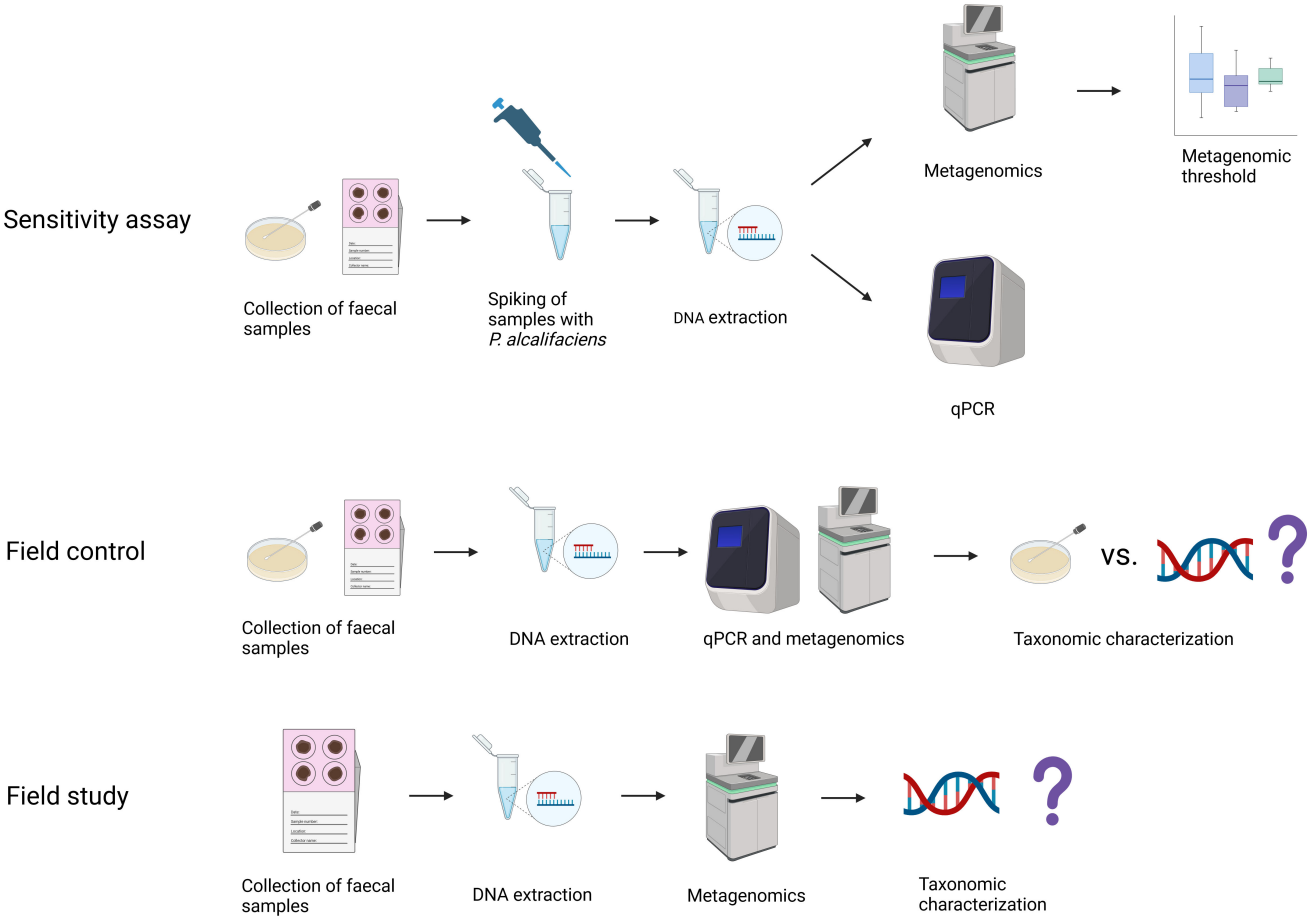
Overview of samples included in the study, the methodology applied, and for which study aims the samples were used. In the sensitivity assay, faecal samples from dogs negative for *Providencia alcalifaciens* by cultivation were spiked with increasing amounts of *P. alcalifaciens*, from 1.1x10^1^ to 1.1x10^5^ CFU, and thresholds for qPCR and metagenomics were established. The field control was used to test the established thresholds on faecal samples naturally contaminated with *P. alcalifaciens*, where samples from dogs positive and negative for *P. alcalifaciens* taken at different timepoints were compared using qPCR, metagenomics, and cultivation. Finally, the field study used the metagenomic abundance threshold to find the occurrence of *P. alcalifaciens* in a large population of dogs. Created with BioRender.com.

This study consisted of three parts: a sensitivity assay, a field control, and a field study (**Figure 1**).

The sensitivity assay aimed to determine the detection limit for *P. alcalifaciens* in dog faeces using shotgun metagenomics. Dog faecal samples were spiked with increasing quantities of *P. alcalifaciens*, and the presence of *P. alcalifaciens* was assessed by qPCR and shotgun metagenomics. The detection limit for both qPCR and metagenomics was determined based on the spiked quantities of *P. alcalifaciens*.

To validate the ability of the detection limit and abundance threshold to detect *P. alcalifaciens* in naturally contaminated dog faecal samples, we analysed culture-positive and culture-negative faecal samples from dogs using shotgun metagenomics and compared the results with cultivation status and CFU levels estimated by qPCR.

Lastly, to generate knowledge on the occurrence of *P. alcalifaciens* in the core microbiota in canine faeces, its occurrence in a large population of healthy dogs was investigated with shotgun metagenomics using the abundance threshold established in the sensitivity assay and confirmed in the field control.

### Materials

#### Collection of faecal samples for the sensitivityassay

Faecal samples from two healthy dogs were collected on TRANSWAB^®^ Amies charcoal swabs (cat. no. MW171, MWE, UK) and faecal cards (LipiDx, Oslo, Norway) (**Table 1**). These faecal cards consist of two sample fields of cellulose matrix (3.5x1.7 cm each), that conserve DNA and RNA at ambient temperatures after drying. Faecal samples from each dog were subjected to cultivation for the presence of *P. alcalifaciens* (see below). For collection on faecal cards, faecal material was stirred prior to applying a thin layer of faeces on the two paper fields of the faecal cards and leaving them to dry for at least two hours (hereafter designated the DFC method). Subsequently, the DFCs were transported to the laboratory and stored at -80°C until DNA extraction (see below).

**Table 1:** Overview of samples, sampling methods, time of sampling and study aim for the different parts of the study.

	Individuals (n)	Total number of samples (n)	Collection method	Time of sampling	Study aim
**Sensitivity assay**	2	2*	Charcoal swab DFC	Autumn 2021	Test sensitivity of metagenomic assay and establish a metagenomic threshold for presence of *P. alcalifaciens*
**Field control**	5	12	Charcoal swab DFC	Autumn 2020 Spring 2021 Autumn 2021	Test metagenomic threshold on samples positive and negative for *P. alcalifaciens* by cultivation
**Field study**	448	448	DFC	2018–2019	Use metagenomic threshold to find occurrence of *P. alcalifaciens* in a large population of dogs

* Several DFCs from each dog were collected at the same timepoint and used to create samples spiked with *Providencia alcalifaciens* at five different dilutions. DFC: dried faecal card. Charcoal swabs were used for cultivation, while DFCs were used for qPCR and metagenomics.

#### Collection of faecal samples for the field control

Owners of five dogs collected faecal samples at two or three timepoints (autumn 2020, spring 2021 and autumn 2021) using both charcoal swabs and the DFC method (**Table 1**). The samples were kept at room temperature and shipped to the laboratory. The swabs were cultured for the presence of *P. alcalifaciens* (as described below) upon arrival at the laboratory, while the faecal cards were stored at -80°C until DNA extraction. To be included in the study, at least one sample per dog had to be positive for *P. alcalifaciens* by cultivation.

#### Collection of datasets for the field study

Shotgun metagenomic datasets from faeces of healthy dogs were acquired through the HUNT One Health project (Norwegian Veterinary Institute, 2023). HUNT One Health provides datasets from stool DFCs collected by animal owners in Trøndelag between 2018 and 2019. DNA extraction and sequencing were conducted by the HUNT One Health project. Each dataset was accompanied by metadata on the dogs’ demographics and health. We included dogs that fulfilled the following inclusion criteria: 1) no history of antibiotics usage during the last 12 months prior to sampling, 2) no medication nor diarrhoea at time of sampling, 3) no reports of prior health issues, and 4) owner-perceived health of the dog as “good” (“god”) or “very good” (“svært god”) at time of sampling, resulting in 448 dogs included (**Table 1**).

### Methods

#### Cultivation of faecal swabs

Faecal swabs in the sensitivity assay and field control were screened for the presence of *P. alcalifaciens* by cultivation; the swabs were streaked directly on bromothymol-blue lactose sucrose (BBLS) agar plates containing polymyxin B (250 000 units/l) (PMB plates) and incubated at 35°C for 24 hours. In addition, all faecal swabs were transferred to 5 ml buffered peptone water (BPW) and incubated at 35°C for 24 hours before 1 µl enriched BPW was plated on PMB plates using an L-shaped spreader and incubated as described earlier. The resulting colonies were quantified by eye (sparse, moderate, rich growth) and described. Field control faecal swabs were in addition routinely streaked on two blood agar plates (one incubated aerobically (5% CO_2_) at 37°C for 24 hours and one anaerobically at 35°C for 48 hours) and on bromothymol-blue lactose (BBL) or BBLS agar plates without polymyxin B. Non-lactose fermenting colonies from PMB plates (sensitivity assay and field control) and BBL/BBLS plates (field control) were subcultured on blood agar plates. Isolates suspected as *P. alcalifaciens* (based on characteristics) were subjected to matrix-assisted laser desorption/ionization–time-of-flight (MALDI-TOF) mass spectrometry (MS) on VITEK^®^ MS (bioMérieux, Marcy l’Étoile, France) for confirmation.

#### Preparation of the spiking strain and DNA extraction from DFCs in the sensitivity assay and field control

An in-house *P. alcalifaciens* strain (2019-04-27799-1-2) (Jørgensen et al., 2021; Haverkamp et al., 2022) was used to spike samples in the sensitivity assay, and the dilutions of this *P. alcalifaciens* strain were prepared the following way: cultivation overnight at room temperature under aerobic conditions in brain heart infusion (BHI) media (cat. no. 11708872, BD Difco), and preparation of a ten-fold dilution series of concentrations from 3.2x10^2^ to 3.2x10^6^ CFU/ml. Bacterial concentration was calculated by serial dilution. Appropriate dilutions were stored at -80°C until use. DNA was extracted from DFCs using the same protocol as in the HUNT One Health project, i.e. the QIAsymphony PowerFecal Pro DNA Kit (cat. no. 938036, Qiagen) on QIAsymphony SP (Qiagen) using the manufacturer’s instructions with modifications. Briefly, four 8 mm circles were aseptically cut from each DFC using a biopsy punch (cat. no. 273693, Kruuse, Norway) and placed in 2 ml PowerBead Pro tubes (cat. no. 19301, Qiagen). Eight hundred µl buffer CD1 were added to the samples, and for samples in the sensitivity assay, 1.1x10^1^ to 1.1x10^5^ CFU of *P. alcalifaciens* were added. All samples were homogenized on FastPrep-24™ Classic (6 rounds of 60 seconds at 6 m/s), and a proteinase K treatment step was included (30 µl proteinase K and incubation for 15 min at 56°C). The supernatant (400–600 µl) was then transferred to a clean 2 ml tube, and the DNA was extracted using QIAsymphony SP. Resulting DNA yields were measured using Qubit. Purity was assessed using NanoDrop ratios 260/230 and 280/230. Mocks and blank controls were included in each extraction round, consisting of clean faecal cards with and without 75 µl ZymoBIOMICS Microbial Community Standard II (cat. no. D6310, Zymo Research), respectively.

#### qPCR

Following the establishment of the qPCR efficiency using a dilution series of *P. alcalifaciens* without faecal material, quantitative detection of *P. alcalifaciens* in DNA extracted from DFCs in the sensitivity assay and field control was performed on the AriaMx platform (Agilent, Santa Clara, CA, USA). All qPCRs were run in three technical replicates with a total reaction volume of 15 µl each consisting of 1X PowerUp™ SYBR™ Green Master Mix (cat. no. A25742, Thermo Fisher Scientific), 200 nM forward primer (38F: TCTGCACGGTGTGGGTGTT), 200 nM reverse primer (110R: ACCGTCACGGCGGATTACT) (Fukushima et al., 2009), and 2 µl template. The qPCR cycling programme was 50°C for 2 min, 95°C for 10 min, 40 cycles of denaturing at 95°C for 15 sec and annealing at 60°C for 1 min, ending with 95°C for 30 sec, 65°C for 30 sec and 95°C for 30 sec. Cq values and melting curves were obtained from amplification plots using Agilent Aria 1.8 software (Agilent, Santa Clara, CA, USA). Replicates that obtained Cq values and specific melting curves were considered positive, and samples were considered positive if at least two of three technical replicates obtained a Cq value. Genome equivalents were calculated based on the standard curve for each run. The lowest detectable number of genome equivalents was 2.1 per reaction, equivalent to 115.8 genome equivalents per sample.

#### Library preparation and shotgun metagenomic sequencing

Library preparation and shotgun metagenomic sequencing were performed by BGI Tech Solutions (Hong Kong) and was identical to the one in the HUNT One Health project. In short, DNA concentration and fragmentation were measured using Qubit and agarose gel electrophoresis. The DNA was subjected to library preparation using the ThruPLEX DNA-Seq Kit (cat. no. R400674, Takara), and resulting libraries were sequenced on the NovaSeq 6000 platform (Illumina, San Diego, CA, USA), yielding paired-end (PE) reads of 150 bp.

#### Data quality control and trimming

**Figure 2:**
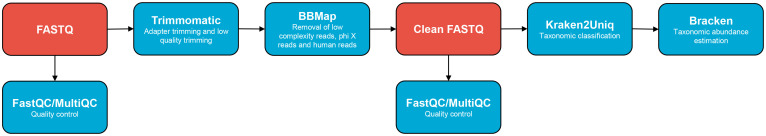
Bioinformatic pipeline used in this study, based on Talos (Haverkamp, 2020).

Clean reads were provided by BGI after processing through their SOAPnuke software to remove adapters, low quality reads and high N base ratio reads (Chen et al., 2018). Our bioinformatic analyses were based on the Talos pipeline for shotgun metagenomic analysis (Haverkamp, 2020) (**Figure 2**) and were performed on the high-performance computer cluster HUNT Cloud (NTNU, 2023). FastQC (v0.11.9) (Andrews, 2023) and MultiQC (v1.10.1) (Ewels et al., 2016) with standard settings were used for quality control. Trimmomatic (v0.39) (Bolger et al., 2014) was used for adapter trimming and low quality trimming with the settings ILLUMINACLIP : TruSeq3-PE.fa:2:30:10, SLIDINGWINDOW:4:15, MINLEN:40, LEADING:3 and TRAILING:3. BBMap (v38.90) (Bushnell, 2014) was used for removal of low complexity reads, phi X reads (accession number: NC_001422.1) and human reads (masked version of human reference HG19: https://drive.google.com/file/d/0B3llHR93L14wd0pSSnFULUlhcUk/edit?usp=sharing).

#### Taxonomic characterization of metagenomic datasets

Kraken2Uniq (v2.1.3) (Breitwieser et al., 2018; Wood et al., 2019; Lu et al., 2022) was used for taxonomic classification to identify *P. alcalifaciens*. KrakenUniq is a fast *k*-mer-based metagenomic classifier that assesses read coverage of each reported reference species in the dataset using unique *k-*mer counts. False-positive reads typically only match a small portion of low complexity regions of the reported reference species’ genome. Such samples will have a low ratio between unique *k-*mer counts and reads for a reported species. Kraken2Uniq integrates the *k*-mer count from KrakenUniq into Kraken2. Kraken2Uniq was run for all samples using the standard database from 2023-03-14 in paired-end mode and with minimum base quality set to 20, minimum hit groups set to 3 and with minimizer data reported. The relative abundances were estimated using Bracken (v2.8) (Lu et al., 2017). Bracken was run in standard mode at species level.

All metagenomic datasets were accompanied by blanks, and the field study samples were in addition accompanied by mocks. If the blank and/or mock samples deviated from expected, the associated metagenomic datasets were removed from the material. Metagenomic datasets with fewer than 10 million PE clean reads were also excluded.

#### Statistics and graphics

We established a metagenomic abundance threshold for *P. alcalifaciens* using two key parameters: unique *k*-mer (U*k*-mer) counts and the ratio of unique *k*-mers to reads (U*k*-mers/reads ratio). The detection limit was established in the sensitivity assay, and corresponding values for both parameters were used as our metagenomic abundance threshold first on the samples from the field control, and then to investigate the occurrence of *P. alcalifaciens* in the field study. The confidence interval for the occurrence of *P. alcalifaciens* was calculated based on sensitivity and specificity of the metagenomic assay in the field control as described by Rogan and Gladen (1978) using an online calculator (Kohn and Senyak, 2023).

Descriptive statistics for U*k*-mer counts and U*k*-mers/reads ratios over spiked-in CFU were generated using STATA/MP (v17.0) (StataCorp LLC, Texas, USA). U*k-*mer counts were log-transformed when necessary. To define the detection limit in the sensitivity assay, the lowest amount of spiked-in CFU of *P. alcalifaciens* with U*k-*mer counts and U*k-*mers/reads ratios significantly higher than those achieved for negative samples was determined using the two-sample t-test for normally distributed data and Wilcoxon rank-sum test for non-parametric data using STATA/MP (v17.0). The distribution of U*k-*mer counts and U*k*-mers/reads ratios over spiked-in CFU were visualized in box- and scatterplots using R (v4.3.1) (R Core Team, 2023) in RStudio (v2023.06.2 + 561) (Posit Team, 2023) and the packages ggplot2 (v3.4.3) (Wickham, 2016), readxl (v1.4.3) (Wickham and Bryan, 2023), ggpmisc (v0.5.4-1) (Aphalo, 2023) and ggtext (v0.1.2) (Wilke and Wiernik, 2022).

Linear regression analysis was used to characterize the correlation between spiked-in CFU and Cq values from qPCR, and spiked-in CFU and genome equivalents as determined by qPCR in the sensitivity assay, using R as described above. The Cq values were thereafter used to calculate the estimated CFU of *P. alcalifaciens* in the field control samples to validate the detection limit suggested in the sensitivity assay. Here, U*k*-mer counts and U*k*-mers/reads ratios were compared to genome equivalents and cultivation results. 

## Results

### The detection limit of shotgun metagenomic analysis for detection of *P. alcalifaciens*

The metagenomic datasets included in the sensitivity assay returned an average of 22.2 million PE clean reads [SD: 1.9 M; range: 17.6 to 23.7 M] for downstream analyses. Log values of U*k-*mer counts were normally distributed (skewness and kurtosis test, *P* = 0.44), while the U*k-*mers/reads ratios and U*k-*mer counts were not (*P =* 0.0194 and *P* = 0.0001, respectively). U*k*-mers classified as *P. alcalifaciens* ranged from 22 to 136 089, while U*k-*mers/reads ratios ranged from 0.3 to 27.2. Both parameters were positively correlated with the spiked-in CFU amounts (**Supplementary Table 1**).

**Figure 3:**
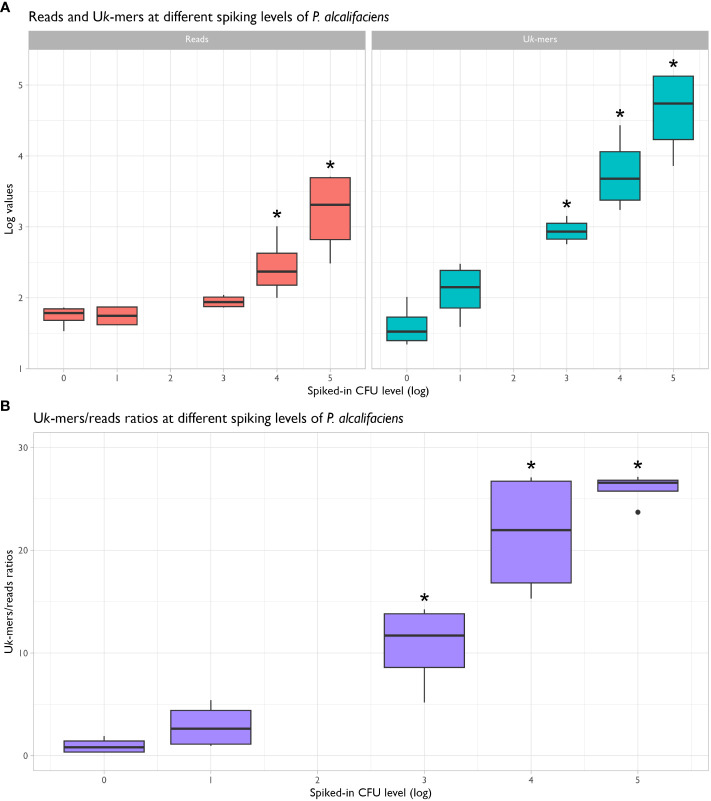
Box-and-whisker plots showing the distribution of reads and unique *k*-mer (U*k*-mer) counts **(A)** and U*k*-mers/reads ratios **(B)** for negative and spiked-in samples in the sensitivity assay. U*k*-mer counts and U*k*-mers/reads ratios were significantly different from negatives when 1.1x10^3^ CFU *Providencia alcalifaciens* or more were added to the samples (*P* < 0.05, marked by *).

Stratified on spiked-in CFU level (treated as string variable), no significant differences in the read counts, U*k-*mer counts and U*k-*mers/reads ratios between the non-spiked samples and samples spiked with 1.1x10^1^ CFU *P. alcalifaciens* were observed (**Figures 3A, B** and **Table 2**). Read counts differed significantly from negatives at 1.1x10^4^ CFU, while both U*k*-mer counts and U*k*-mers/reads ratios differed significantly at 1.1x10^3^ CFU. At this spiking level, the corresponding ranges for U*k*-mers classified as *P. alcalifaciens* and U*k*-mers/reads ratios were 569 to 1425 and 5.2 to 14.3, respectively (**Table 3**). Based on this, the suggested metagenomic threshold chosen in the downstream analyses was a U*k-*mer count of 569 and a U*k-*mers/reads ratio of 5.2.

**Table 2:** U*k*-mer counts, U*k*-mer counts (log) and U*k*-mers/reads ratios are statistically significantly higher in spiking levels from 1.1x10^3^ CFU *Providencia alcalifaciens* and above compared to negative samples.

Parameter	Groups tested over spiked-in CFU (log) using two-tailed t-test or two-sample Wilcoxon rank-sum (Mann–Whitney) test			
	CFU log 0 vs. 1	CFU log 0 vs. 3	CFU log 0 vs. 4	CFU log 0 vs. 5
U*k*-mer counts	*P* = 0.20	*P* = 0.029	*P* = 0.029	*P* = 0.029
U*k*-mer counts (log)*	*P* = 0.099	*P* = 0.0003	*P* = 0.0004	*P* = 0.0001
U*k*-mers/reads ratios	*P* = 0.34	*P* = 0.029	*P* = 0.029	*P* = 0.029
Read counts	*P* = 0.69	*P* = 0.057	*P* = 0.029	*P* = 0.029

The absolute number of reads *P. alcalifaciens* was significantly higher in samples that were spiked with 1.1x10^4^ CFU *P. alcalifaciens* and more. U*k*-mer: unique *k*-mer. * Denotes that the parameter was assessed using the two-tailed t-test, while the other parameters were assessed using the Wilcoxon rank-sum (Mann–Whitney) test.

**Table 3:** The qPCR and metagenomics results for the sensitivity assay, where culture-negative faecal samples were spiked with increasing amounts of *Providencia alcalifaciens*.

*P. alcalifaciens* added	Dog A			Dog B			Medians for both dogs (range)		Empty faecal card		
CFU	Detected/total	Genome equivalents avg.	Reads (%)	Detected/total	Genome equivalents avg.	Reads (%)	U*k*-mer counts	U*k*-mers/reads ratio	Detected/total	Genome equivalents avg.	Reads (%)
**0 (n=5)**	0/3	<115.8	R1: 69 (0.0007%) R2: 73 (0.0007%)	0/3	<115.8	R1: 54 (0.0005%) R2: 34 (0.0004%)	34.5 (22, 103)	0.8 (0.3, 1.9)	0/1	<115.8	41 (0.02%)
**1.1x10^1^ (n=4)**	0/3	<115.8	R1: 41 (0.0005%) R2: 75 (0.0007%)	1/2	<115.8	R1: 42 (0.0004%) R2: 74 (0.0008%)	157.5 (39, 301)	2.6 (1.0, 5.4)	0/2	<115.8	NA
**1.1x10^2^ (n=4)**	0/3	<115.8	NA	1/3	<115.8	NA	NA	NA	0/2	<115.8	NA
**1.1x10^3^ (n=4)**	2/3	137.7 (SD: 79.8)	R1: 110 (0.001%) R2: 73 (0.0008%)	3/3	225.3 (SD: 79.8)	R1: 76 (0.0008%) R2: 100 (0.001%)	874.5 (569, 1425)	11.7 (5.2, 14.3)	2/2	<115.8	NA
**1.1x10^4^ (n=4)**	3/3	413.7 (SD: 287.6)	R1: 100 (0.001%) R2: 173 (0.002%)	3/3	2901.0 (SD: 2654.6)	R1: 318 (0.003%) R2: 1020 (0.01%)	5632 (1733, 27 140)	22.0 (15.3, 27.1)	2/2	131.4 (SD: 37.1)	NA
**1.1x10^5^ (n=4)**	3/3	496.1 (SD: 375.4)	R1: 305 (0.003%) R2: 857 (0.008%)	3/3	23 168.1 (SD: 5067.7)	R1: 5094 (0.05%) R2: 4886 (0.05%)	77 674, (7232, 136 089)	26.6 (23.7, 27.2)	2/2	2154.0 (SD: 335.9)	NA

NA: not applicable (not sequenced). R1: biological replicate 1. R2: biological replicate 2. Read numbers denote reads assigned to *P. alcalifaciens* by Kraken2Uniq, while relative abundances in percent denote relative abundances estimated by Bracken. U*k*-mer: unique *k*-mer.

**Figure 4:**
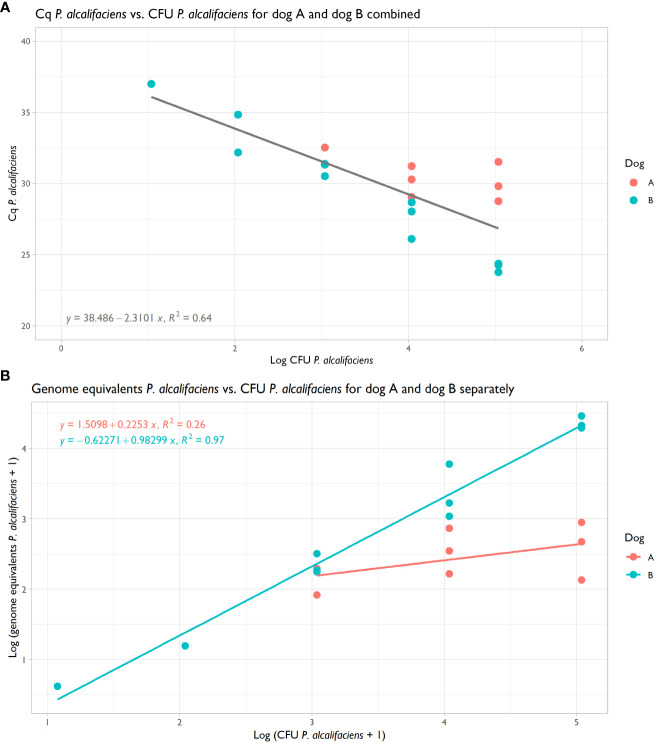
**(A)** Scatterplot of Cq *Providencia alcalifaciens* from qPCR vs. CFU *P. alcalifaciens* for dog A and B, with regression showing a shared correlation coefficient R^2^ of 0.64. **(B)** Scatterplot of genome equivalents *P. alcalifaciens*, as estimated from Cq values, vs. CFU *P. alcalifaciens* for dog A and B, with regressions showing correlation coefficients R^2^ of 0.26 and 0.97, respectively.

The qPCR was highly efficient and could detect 2.1 genome equivalents of *P. alcalifaciens* in pure DNA (**Supplementary Figure 1**). This qPCR was employed to quantify the abundance of *P. alcalifaciens*, facilitating comparison with the metagenomic datasets. Cq values and their corresponding genome equivalents correlated well with CFU introduced into the samples, although variability was observed between the two dogs (**Figures 4A, B**). Notably, for dog B, there was a pronounced correlation between spiked CFU and genome equivalents (R^2^: 0.97; **Figure 4B**), whereas for dog A, the correlation was more modest (R^2^: 0.26; **Figure 4B**). The minimum spiking level detectable was 1.1x10^3^ CFU, which yielded 138 genome equivalents in dog A and 225 in dog B, corresponding to a Cq value of 31.4 (**Table 3**).

### Field control

Faecal material from five dogs were sampled on two and three occasions, returning 24 samples collected on swabs (n=12) and DFCs (n=12). The samples were analysed by cultivation, qPCR, and shotgun metagenomics for the occurrence of *P. alcalifaciens*. Cultivation yielded 41.7% (n=5/12) faecal samples positive for *P. alcalifaciens* (**Table 4**). In two of the samples, the growth on plates was sparse, while one had moderate, and one had rich growth of *P. alcalifaciens.* One sample was positive only after enrichment. Taking the detection limit established from the spiked samples into consideration, only two of the five samples found positive by cultivation (40%) were deemed positive by qPCR. These samples, namely 49_H1 (2580 genome equivalents) and 56_H3 (20 074 genome equivalents), had moderate and rich growth of *P. alcalifaciens* on direct plating, respectively.

**Table 4:** Comparison of cultivation status for *Providencia alcalifaciens*, qPCR and metagenomics for the field control.

Sample	Dog	Sampling time	Cultivation		qPCR			Metagenomics		
			Before enrichment	After enrichment	Detected/total	Cq avg.	Genome equivalents	Reads (%)	U*k*-mer counts (log)	U*k-*mers/reads ratios
**49_H1**	1	Autumn 2020	Positive: moderate growth	NA	3/3	27.4 (SD: 0.3)	2580.4	1812 (0.02%)	39 232 (4.6)	21.7
**50_H1**	1	Spring 2021	Negative	Negative	1/3	No Cq	<115.8	153 (0.002%)	193 (2.3)	1.3
**51_H2**	2	Autumn 2020	Negative	Positive	1/3	No Cq	<115.8	101 (0.002%)	309 (2.5)	3.1
**52_H2**	2	Spring 2021	Negative	Negative	1/3	No Cq	<115.8	67 (0.001%)	218 (2.3)	3.3
**53_H2**	2	Autumn 2021	Negative	Negative	0/3	No Cq	<115.8	57 (0.0006%)	107 (2.0)	1.9
**55_H3**	3	Spring 2021	Negative	Negative	2/3	34.9 (SD: 1.7)	<115.8	57 (0.0007%)	148 (2.2)	2.6
**56_H3**	3	Autumn 2021	Positive: rich growth	NA	3/3	24.4 (SD: 0.0)	20 073.7	8584 (0.08%)	190 208 (5.3)	22.2
**61_H5**	5	Spring 2021	Negative	Negative	0/3	No Cq	<115.8	74 (0.002%)	113 (2.1)	1.5
**62_H5**	5	Autumn 2021	Positive: very sparse growth	NA	0/3	No Cq	<115.8	73 (0.001%)	165 (2.2)	2.3
**63_H6**	6	Autumn 2020	Negative	Negative	0/3	No Cq	<115.8	71 (0.0007%)	104 (2.0)	1.5
**64_H6**	6	Spring 2021	Negative	Negative	3/3	32.1 (SD: 0.2)	<115.8	31 (0.0003%)	56 (1.7)	1.8
**65_H6**	6	Autumn 2021	Positive: sparse growth	NA	3/3	34.3 (SD: 0.4)	<115.8	25 (0.0002%)	87 (1.9)	3.5

Read numbers denote reads assigned to *P. alcalifaciens* by Kraken2Uniq, while relative abundances in percent denote relative abundances estimated by Bracken. Coloured cells indicate that the parameter exceeds threshold set. NA: not applicable. U*k*-mer: unique *k*-mer. Threshold used: 569 U*k*-mer counts and a U*k*-mers/reads ratio of 5.2. qPCR was run in triplicates.

**Figure 5:**
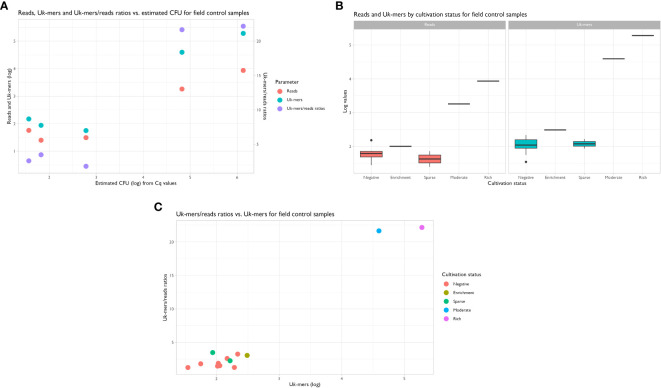
**(A)** Scatterplot showing the distribution of reads, unique *k*-mers (U*k*-mers) and U*k*-mers/reads ratios vs. estimated CFU. Samples with estimated CFU > 3 logs for *Providencia alcalifaciens* had higher U*k*-mer counts and U*k*-mers/reads ratios than the samples with no CFU or low amounts of *P. alcalifaciens*. Linear regression used: Cq = 38.5−2.3xCFU (**Figure 4A**). **(B)** Box-and-whisker plots showing the distribution of reads and U*k*-mers vs. cultivation status. **(C)** Scatterplot showing the relationship between U*k*-mers/reads ratios vs. U*k*-mers (log) by cultivation status. Samples with moderate and rich growth of *P. alcalifaciens* cluster together with high U*k*-mers (log) and U*k*-mers/reads ratios, separating clearly from samples with sparse and no growth.

The metagenomic data from the samples in the field control returned an average of 21.9 million PE clean reads [SD: 1.7 M; range: 19.2 to 23.7 M] for downstream analyses. Using the suggested abundance threshold from the sensitivity assay, i.e. a U*k-*mer count of 569 and a U*k-*mers/reads ratio of 5.2, the same two samples positive by qPCR were also positive by metagenomics. Sample 49_H1 had 39 232 U*k-*mers reported as *P. alcalifaciens* and a U*k-*mers/reads ratio of 21.7, while sample 56_H3 had 190 208 U*k-*mers classified as *P. alcalifaciens* with a U*k-*mers/reads ratio of 22.2 (**Figures 5A–C** and **Table 4**). The numbers of reads mapping to *P. alcalifaciens* for 49_H1 and 56_H3 were 1812 (relative abundance: 0.02%) and 8584 reads (0.08%), respectively. Stool samples from the same dogs collected on separate occasions that were negative for *P. alcalifaciens* by cultivation and qPCR, returned 153 (0.002%) and 57 (0.0007%) reads mapped to *P. alcalifaciens* and U*k-*mer counts and U*k-*mers/reads ratios well below the suggested abundance threshold (**Table 4**). Considering just cultivation results, samples with sparse growth of *P. alcalifaciens* could not be separated from negative samples based on U*k-*mer counts (**Figures 5B, C**). Using CFU estimated from Cq values, negative and positive samples could be separated on U*k-*mer counts and U*k*-mers/reads ratios when the samples contained more than 1.1x10^3^ CFU of *P. alcalifaciens* (**Figure 5A**). Therefore, the suggested detection limit for both qPCR and metagenomics from the sensitivity assay was valid for naturally contaminated samples in the field control.

We thereafter compared the sensitivity and specificity of the metagenomic abundance threshold for *P. alcalifaciens* and qPCR with bacterial cultivation. Cultivation yielded 41.7% positive samples, while both qPCR and metagenomics yielded 16.7% positive samples each (**Table 4**). Using cultivation as gold standard, qPCR and metagenomics had a test sensitivity of only 40% (n=2/5), leaving three of five culture-positives as false negatives. None of the samples were positive only by qPCR or metagenomics, yielding a specificity of 100%. The positive predictive value was therefore 100%, while the negative predictive value was 70%.

### Field study

Using the metagenomic abundance threshold of 569 U*k*-mers and a U*k*-mers/reads ratio of 5.2, we investigated the occurrence of *P. alcalifaciens* in metagenomic datasets of faecal material from healthy dogs received from the HUNT One Health project.

After quality control, datasets from 362 dogs remained (**Data Sheet 2**), with an average of 20.0 million PE clean reads [SD: 3.5 M; range: 10.0 to 23.8 M] for downstream analyses. According to our abundance threshold, four of 362 (1.1%, 95% CI [0.0, 6.8]) faecal samples were positive for *P. alcalifaciens*, namely DNA_H1H_14_B1, DNA_H1H_26_B3, DNA_H1H_18_D3 and DNA_H1H_23_F3 (**Data Sheet 2**). The bacterium was identified in low read numbers by Kraken2Uniq (median: 3781; range: 128 to 23 971), with a median estimated number of U*k*-mers assigned to *P. alcalifaciens* at 83 548.5 (range: 967 to 340 549). The relative abundance of *P. alcalifaciens* estimated by Bracken was low (median: 0.04%; range: 0.00 to 0.81%).

## Discussion

Here, we established the detection limit for a metagenomic assay and set a metagenomic abundance threshold for *P. alcalifaciens* in faecal samples, with the goal of investigating the occurrence of this bacterium in the faecal microbiota of healthy dogs. The detection limit for qPCR and shotgun metagenomics was 1.1x10^3^ CFU *P. alcalifaciens* in faecal samples smeared on DFCs, which corresponded to 225 genome equivalents and 569 U*k*-mers. The occurrence of *P. alcalifaciens* above this detection limit in healthy dog faeces was low (upper confidence at 6.8%), but a higher occurrence of this bacterium in our dataset at a lower abundance cannot be excluded.

Determining an abundance threshold for the detection of microbiological taxa in metagenomic samples is challenging and depends on several factors, such as reference database, study design, sampling material, sequencing effort, and sample complexity. For instance, Lindstedt et al. (2022) highlighted that the rate of false positives is dependent on the abundance of related bacteria in the sample, pointing to the misclassification of query reads to target bacteria in the database. Further, McIntyre et al. (2017) report that *k-*mer-based methods, such as Kraken2Uniq, have a positive relationship between sequencing depth and misclassified reads. Therefore, an abundance threshold is needed, and several studies have attempted to define abundance thresholds using different approaches. Miller et al. (2019) set a threshold for bacteria, fungi, and parasites in cerebrospinal fluid samples based on the ratio of relative abundances in samples and blank controls. Using receiver operating characteristic (ROC) curves, they found that the accuracy of organism detection was maximized when this ratio was 10. A study on *Campylobacter* in air of poultry houses used a threshold of 50 reads per sample based on the number of putative false *Campylobacter* reads in mock samples (Haverkamp et al., 2021), whereas a study on *Klebsiella* in human faecal samples suggested an abundance threshold of 0.01% to accurately estimate its occurrence based on spiked microbiomes (Lindstedt et al., 2022). We choose to use unique *k*-mer counts as parameter for metagenomic abundance threshold in place of read counts as the former have improved species-recall and better separation between negative and positive samples (Breitwieser et al., 2018), increasing the robustness of the metagenomic assay. Further, we achieved a lower detection limit by unique *k*-mer counts than by absolute read numbers, as the latter showed statistical significance at spiking level 1.1x10^4^ CFU rather than 1.1x10^3^.

Our metagenomic abundance threshold of 569 U*k*-mers had a detection limit of 1.1x10^3^ CFU per sample for the metagenomic assay. Our threshold is lower than the recommendations from the authors of KrakenUniq, who suggest a pathogen detection threshold of 2000 U*k*-mers per million sequenced reads (Breitwieser et al., 2018), i.e. 80 000 U*k-*mers for our dataset. Even so, the achieved detection limit of 1.1x10^3^ CFU is high, and sensitivity of the metagenomic assay was low. We pushed the detection limit and metagenomic threshold as low as possible to increase sensitivity. However, a lower threshold would increase the number of spurious classifications of positive and negative samples dramatically. Using qPCR, the detection limit was equally high, even though the qPCR assay could detect as few as 2.1 genome equivalents for pure *P. alcalifaciens* DNA. As a result, only 40% of samples in the field control positive by cultivation were positive by qPCR and metagenomics, indicating that cultivation was a more sensitive method for detection of *P. alcalifaciens* in stool. This is in line with the results found by Andersen et al. (2017B), where a best-case detection limit for *Campylobacter* in human faecal samples was 7.75x10^4^ CFU/ml. Contrary to our findings, Liu et al. (2018) found that a higher sensitivity for *Shigella* in human faecal samples was obtained by qPCR and metagenomics than by cultivation. An explanation could be that *Shigella* is better represented in the databases than *P. alcalifaciens*, resulting in a higher proportion of *Shigella* reads being correctly identified. Andersen et al. (2017A) found that qPCR and metagenomics were not always in agreement, and a spiking level of 10^3^ CFU/g *C. jejuni* in human faecal samples was positive by Kraken, but negative by qPCR, due to sample preparation.

Faeces as matrix have a high level of background noise due to their complex composition of microbes compared to sterile or low microbial load samples (de Goffau et al., 2018). The complexity of the sample can affect the detection of low-abundance bacteria since the presence of more abundant species may overshadow the signal from rare species. It might therefore be easier to identify rare species in low microbial load samples. Further, it might be that the DNA extraction protocol was not well suited to extract *P. alcalifaciens* from dog faeces. Our qPCR was able to detect 2.1 genome equivalents per reaction in pure DNA, but in faecal samples it was only able to achieve a positive signal in samples spiked with 1.1x10^3^ CFU and above. Albeit the mock communities were as expected, we cannot rule out that the extraction efficiency of low-abundance microbes such as *P. alcalifaciens* was not optimal in a highly complex matrix such as faeces.

In the field control, we have assumed that cultivation status represents the true presence or absence of *P. alcalifaciens*. It is possible that this is incorrect, and that the bacterium is present in some of the samples negative by cultivation. This would mean that the bacterium is present in samples with lower numbers of U*k-*mers identified as *P. alcalifaciens* and that our metagenomic threshold should have been even lower. Some bacteria enter a viable but non-culturable (VBNC) state under certain conditions (Fakruddin et al., 2013) and may therefore only be detected by e.g. molecular methods. However, the qPCR had the same detection limit. Further, *P. alcalifaciens* is not a fastidious organism, and is readily recoverable by cultivation after long-term starvation (Baker et al., 2019). Additionally, the samples we used for cultivation were collected on charcoal swabs which prolong the viability of bacteria, and further, the swabs were pre-enriched in buffered peptone water.

With our low sensitivity, we found that the occurrence and abundance of *P. alcalifaciens* in healthy dogs were low. Four of 362 samples contained more than 1.1x10^3^ CFU *P. alcalifaciens*. Our findings are in line with previous work where no *Providencia* spp. was found by amplicon sequencing of faecal samples from healthy dogs collected in 2017 to 2018 (Herstad et al., 2021). In addition, during the AHDS outbreak in 2019, *P. alcalifaciens* was identified in 11% of healthy dogs in Oslo by cultivation, while only 1.7% of the samples from Bergen and Tromsø were positive (Jørgensen et al., 2021). Both the faecal cards and the metagenomic assay are sensitive for discrepancies in sampling amount and handling, as seen between dog A and B in the sensitivity assay. In the field control and field study, the DFCs were collected by the owners, shipped, and stored before preparation. There are several uncertainties related to this, such as how well the instructions were followed, and the amounts of faecal material collected. It is possible that other collection methods would have created more accurate metagenomic datasets based on more even amounts of input material. The DFC collection method is however gaining increasing evidence as to its ability to preserve DNA and RNA, and several studies have shown that this collection method is suitable for generating metagenomic datasets (Taylor et al., 2017; Botnen et al., 2023). This only applies when the collection method is performed correctly, which could be variable between owners. Our estimate of a low occurrence (upper confidence at 6.8%) of *P. alcalifaciens* in healthy dogs should be viewed as a conservative estimate, encompassing only bacteria present in quantities exceeding 1.1x10^3^ CFU. Moreover, among the four dogs that carried *P. alcalifaciens*, the bacterium was found in low quantities in their faeces. These observations suggest that *P. alcalifaciens* is not a dominant bacterium in the faecal microbiota of dogs. Such a finding lends credence to the hypothesis that this bacterium is associated with AHDS, and a possible aetiology for the outbreak of canine AHDS in 2019.

There were some limitations with our study design. The study would have benefitted from more replicates and finer gradients of spiking *P. alcalifaciens* concentrations in the sensitivity assay. Both would have aided us in establishing a more robust abundance threshold for detection of *P. alcalifaciens*. In addition, we had too few culture-positive faecal samples naturally containing *P. alcalifaciens* to test the suggested threshold on, and three of five had sparse growth or were dependent on enrichment for detection by cultivation. It is however clear that bacterial culture of fresh faecal material is more sensitive than qPCR and metagenomics of DNA extracted from DFCs.

To conclude, metagenomics has the potential to be a powerful diagnostic tool but should not be used uncritically. Our study indicates that shotgun metagenomics performed on faecal samples collected on DFCs can detect low-abundance pathogens, albeit only confidently differentiate between positive and negative samples which contain more than 1.1x10^3^ CFU for our pathogen of interest. More research is needed to obtain satisfying sensitivity and specificity for low-abundance pathogens.

## Data availability statement

The datasets presented in this study can be found in the European Nucleotide Archive (https://www.ebi.ac.uk/ena) at the accession numbers PRJEB66439 and PRJEB66438.

## Ethics statement

According to Norwegian legislation and institutional requirements, the methods used in our animal studies did not require ethical approval. Written informed consent was obtained from the owners for the participation of their animals in this study.

## Author contributions

AA: Data curation, Formal analysis, Funding acquisition, Investigation, Methodology, Software, Visualization, Writing – original draft. EMS: Investigation, Methodology, Writing – review & editing. SN: Conceptualization, Formal analysis, Funding acquisition, Methodology, Supervision, Writing – review & editing. TH: Data curation, Methodology, Software, Supervision, Writing – review & editing. SR: Conceptualization, Funding acquisition, Resources, Supervision, Writing – review & editing. ES: Conceptualization, Funding acquisition, Project administration, Supervision, Writing – review & editing. AL: Conceptualization, Data curation, Funding acquisition, Investigation, Methodology, Project administration, Resources, Software, Supervision, Writing – review & editing.
