# Supplementary material for: Defining a metagenomic threshold for detecting low abundances of Providencia alcalifaciens in canine faecal samples
Source: Front Cell Infect Microbiol. 2024 Feb 28;14:1305742. doi: 10.3389/fcimb.2024.1305742 (PMC10933104; doi:10.3389/fcimb.2024.1305742)
Supplement: Supplementary file 1 [file DataSheet_1.docx]

Supplementary Material

Defining a metagenomic threshold for detecting low abundances of *Providencia alcalifaciens* in canine faecal samples

Anja Maria Aardal, Eiril Moen Soltvedt, Simen Foyn Nørstebø, Thomas H.A. Haverkamp, Sabrina Rodriguez-Campos, Ellen Skancke, Ann-Katrin Llarena*

*** Correspondence:** Corresponding Author: [ann-katrin.llarena@nmbu.no](mailto:ann-katrin.llarena@nmbu.no)

**Supplementary Table 1***.* Regression models describing the relationship between unique *k*-mers (U*k*-mers) and U*k*-mers/reads ratios and spiked-in CFU.

| **Dependent variable** | **Independent variable** | **Model** | **R-value** | **P-value** |
| --- | --- | --- | --- | --- |
| U*k*-mers/reads ratios | Spiked-in CFU (log) | Y = 5.2 x log(CFU+1) – 1.3 | R^2^ = 0.86 | *P* << 0.0001 |
| U*k*-mer counts (log) | Spiked-in CFU (log) | Y = 0.58 x log(CFU+1) + 1.5 | R^2^ = 0.87 | *P* << 0.0001 |
| U*k*-mer counts | Spiked-in CFU | Y = 0.68 x CFU + 708.2 | R^2^ = 0.53 | *P* = 0.0003 |


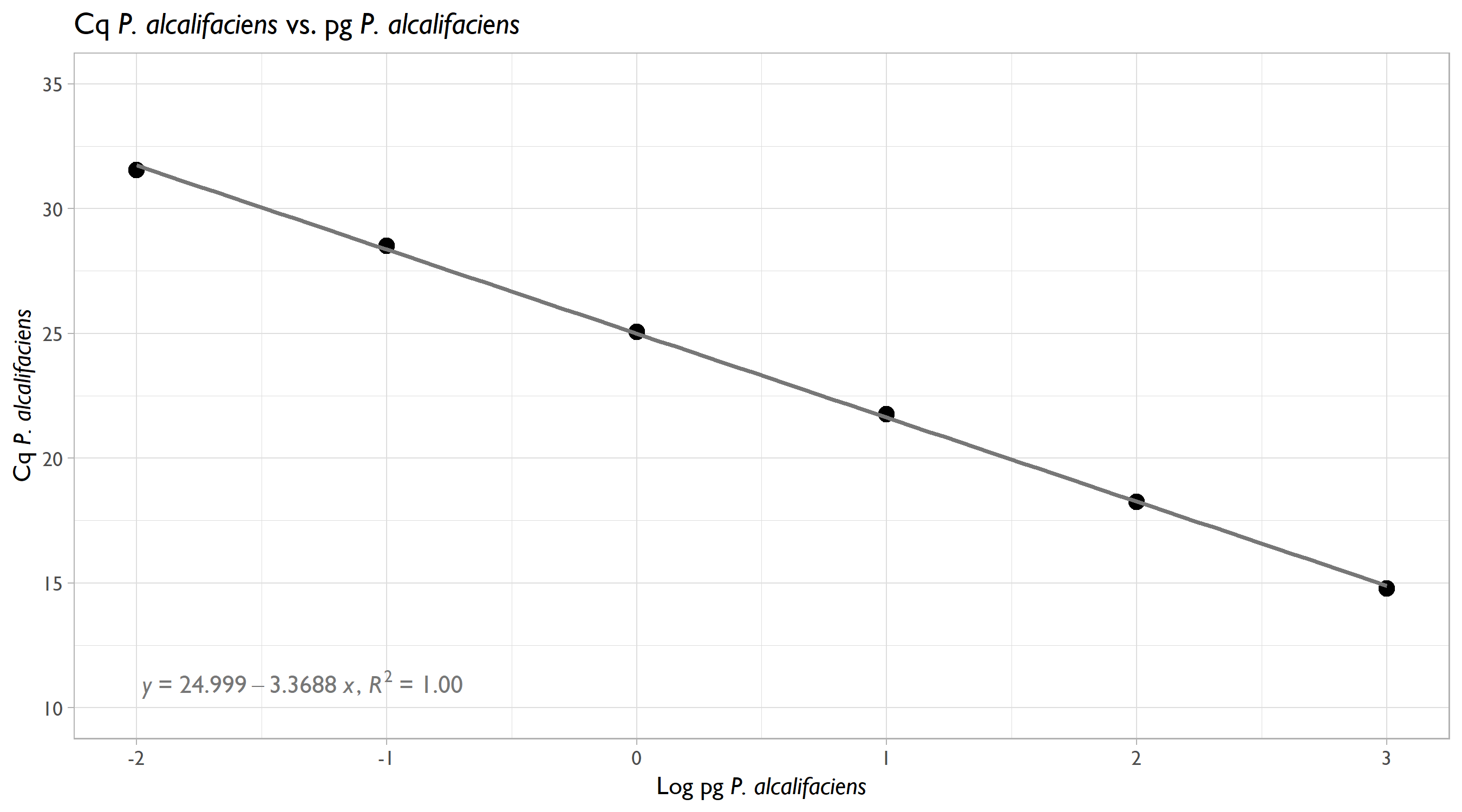


**Supplementary Figure 1.** The efficiency of the qPCR assay without faecal material was 98.1%, and the lowest concentration of DNA detected was 0.01 pg, corresponding to approximately two genome copies.
